# Self-Assembled Hydrogels: A Novel Drug Delivery System for Osteoarthritis

**DOI:** 10.3390/cimb48020211

**Published:** 2026-02-14

**Authors:** Hongjuan Wen, Xintong Gu, Kuo Wen, Weibo Qin, Yiwen Geng, Meilun Wang, Chaoya Yang, Qi Wang, Ning Cui, Da Liu

**Affiliations:** 1 School of Health Management, Changchun University of Chinese Medicine, Changchun 130117, China; wenhj@ccucm.edu.cn; 2 School of Pharmacy, Changchun University of Chinese Medicine, Changchun 130117, China; 24204893104@stu.ccucm.edu.cn (X.G.); 18378026567@163.com (W.Q.); 13844846135@163.com (Y.G.); 18740094882@163.com (M.W.); 15584356629@163.com (C.Y.); 19819514897@163.com (Q.W.); 3 Public Laboratory Centre, Changchun University of Chinese Medicine, Changchun 130117, China; 4 College of Traditional Chinese Medicine, Changchun University of Chinese Medicine, Changchun 130117, China; 15590142627@163.com

**Keywords:** self-assembled hydrogels, osteoarthritis, drug delivery, exosomes, clinical translation

## Abstract

Osteoarthritis (OA) is a prevalent degenerative disease of the musculoskeletal system worldwide. Self-assembled hydrogels, as a novel drug delivery system, have demonstrated significant advantages in the treatment of OA. Through non-covalent interactions such as hydrogen bonding, hydrophobic interactions, electrostatic interactions, and π-π stacking, these hydrogels spontaneously form a three-dimensional network structure under physiological conditions without the need for chemical crosslinking agents, offering excellent biocompatibility, injectability, and controllable degradation properties. This system enables in -situ gelation within the joint, minimally invasive injection, sustained and controlled drug release, and intelligent responsive release. It is suitable for various delivery forms, including single-drug targeted delivery, exosome-based composite synergistic delivery, and microenvironment-responsive precise delivery, effectively inhibiting inflammation and promoting cartilage repair. Despite facing challenges in clinical translation, such as consistency in large-scale production, long-term safety evaluation, and regulatory standards, continued optimization in material design and preparation processes holds promise for self-assembled hydrogels to become a key platform for precise and minimally invasive OA treatment, offering new solutions for joint disease therapy.

## 1. Introduction

Osteoarthritis (OA) is a common degenerative disease of the musculoskeletal system worldwide, mainly affecting joints such as the knees, hands, and hips. It has a high prevalence and is prone to causing functional disability in the elderly, imposing a heavy burden on individuals, families, and society. The etiology of this disease is unclear, its pathogenesis is complex, and the core pathology is the progressive degradation of the extracellular matrix of chondrocytes accompanied by limited cartilage regeneration capacity [1].

**Figure 1:**
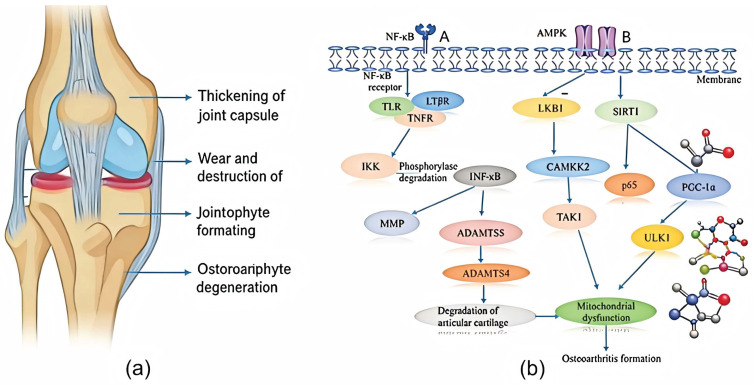
(**a**) Clinical pathological characteristics of osteoarthritis. (**b**) Schematic diagram of the classic pathways of osteoarthritis. A: NF-κB pathway, NF-κB signaling activity in OA chondrocytes. NF-κB signal receptors include TNFR, TLR, LTβR, etc. Activated IKK regulates its phosphorylation to induce IκB degradation. NF-κB signaling induces the secretion of degrading enzymes (such as matrix metalloproteinases MMP, ADAMTS4, and ADAMTS5), while leading to articular cartilage degradation. B: AMPK pathway. In OA chondrocytes, inhibition of AMPK phosphorylation reduces ULK1 expression. At the same time, decreased AMPK phosphorylation also downregulates the expression of PGC-1α in articular chondrocytes through SIRT1, leading to mitochondrial dysfunction and ultimately osteoarthritis. Created with BioGDP.com (accessed on 20 January 2026) [7].

Specifically, the pathogenesis of osteoarthritis is complex and progressive, centered on the abnormal regulation of key signaling pathways such as nuclear factor κB (NF-κB) [2], p38 mitogen-activated protein kinase (p38 MAPK) [3], and Wnt/β-catenin [4]. It is also closely associated with impaired autophagic clearance function of macrophages, imbalanced expression of various transcription factors, and dysregulation of the pro-inflammatory and anti-inflammatory cytokine network. Its core pathological feature is the imbalance between mechanical stress and extracellular factors, and the hallmark pathological change is the progressive degradation of the extracellular matrix of chondrocytes. Moreover, a vicious circle is formed between the degradation of the extracellular matrix and local inflammatory responses. Abnormally activated signaling pathways promote the massive release of pro-inflammatory cytokines such as tumor necrosis factor α (TNF-α), interleukin 1β (IL-1β) [5], and interleukin 6 (IL-6), which further mediate the overexpression of various proteases in articular cartilage and synovial tissue, including matrix metalloproteinase 13 (MMP-13) and a disintegrin and metalloproteinase with thrombospondin motifs 5 (ADAMTS5) [6]. These proteases ultimately degrade and destroy the collagen fiber network and proteoglycans in the articular cartilage matrix, leading to cartilage degeneration. In addition, the ischemic nature of cartilage tissue results in significantly limited self-regeneration capacity after joint injury. Therefore, the timely implementation of tissue repair and inhibition of disease progression is of great clinical significance. To better contextualize these mechanisms, Figure 1 depicts the characteristic structural lesions of an osteoarthritic knee joint, and the molecular signaling cascades that drive cartilage degradation and inflammation.

To overcome the above challenges, the development of new local drug delivery systems capable of achieving long-acting and controllable release of drugs in the joint cavity has become a research frontier and urgent need in the field of OA treatment [8]. Against this background, self-assembled hydrogels, as a highly promising intelligent biomaterial, have attracted widespread attention [9]. Self-assembled hydrogels are semi-solid materials formed by specific small molecules, peptides, or polymers that spontaneously and orderly assemble into a three-dimensional network structure through non-covalent interactions (such as hydrogen bonding, hydrophobic interactions, π-π stacking, and host-guest recognition) under physiological conditions, while entrapping a large amount of water. This unique formation mechanism endows them with numerous excellent properties, including excellent biocompatibility, adjustable biodegradability, a high water-containing microenvironment similar to the natural extracellular matrix, injectability (facilitating minimally invasive delivery), and intelligent responsiveness to physiological stimuli (such as pH, enzymes, and temperature) [10].

Based on these properties, self-assembled hydrogels show great advantages as drug delivery carriers in OA treatment. They can efficiently encapsulate and load therapeutic drugs into the joint cavity [11]. Through the physical barrier and chemical interactions of their three-dimensional network, they significantly slow down the drug release rate and achieve sustained drug delivery for weeks to months. This not only greatly prolongs the local action time of drugs [12], reduces the frequency of administration, but also maintains an effective therapeutic concentration in the joint cavity, improving the curative effect while reducing systemic toxicity. In addition, through molecular design, intelligent hydrogels responsive to the OA pathological microenvironment can be constructed to achieve on-demand and targeted drug release, further enhancing the precision of treatment.

## 2. Formation Mechanisms and Key Properties of Self-Assembled Hydrogels

Self-assembled hydrogels have become ideal carriers for local OA treatment due to their characteristics of no need for external intervention and precise regulation of structure and function. This section systematically elaborates on their formation principles, core properties oriented to OA treatment, and highlights their application advantages through quantitative comparison with traditional hydrogels.

### 2.1. Formation Mechanism of Self-Assembled Hydrogels

**Figure 2:**
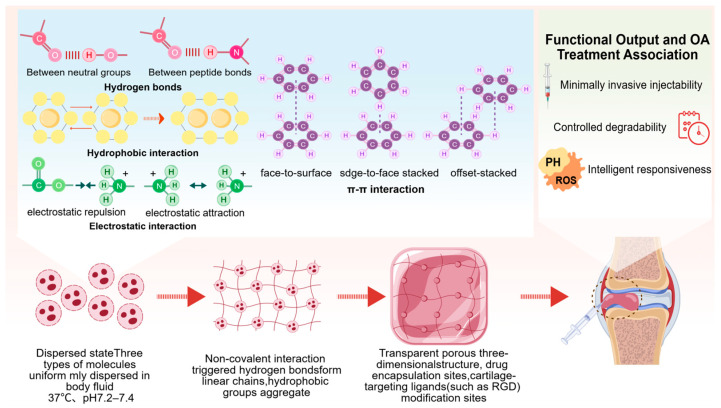
This schematic illustrates the self-assembly mechanism and therapeutic functionality of an intelligent hydrogel for osteoarthritis (OA) treatment. Created with BioGDP.com (accessed on 20 October 2025) [7].

The core formation mechanism of self-assembled hydrogels lies in the spontaneous and ordered physicochemical process of molecular self-assembly, as shown in Figure 2. Essentially, hydrophilic polymer molecules transition from a dispersed state to an ordered aggregated state through intermolecular non-covalent interactions under physiologically friendly and mild conditions (37 °C, the temperature of the human body, and pH 7.2–7.4 of body fluids), ultimately constructing a gel system with a porous three-dimensional network structure [13,14]. This process does not require the involvement of chemical crosslinkers such as glutaraldehyde and genipin, nor the driving force of external physical fields including ultraviolet irradiation, ultrasonic induction, and freeze–thaw cycles [15,16,17]. It can be achieved solely by relying on the structural characteristics of the molecules themselves and their adaptability to the environment [18]. Thus, it possesses both dynamic reversibility and excellent biocompatibility, fundamentally avoiding cytotoxicity or chronic inflammatory reactions caused by residual chemical crosslinkers [19].

The core driving forces of the self-assembly process originate from four types of non-covalent interactions. These forces do not exist in isolation but jointly regulate the formation, stability, and functionality of the gel network in a synergistic and complementary manner [20]. As one of the core driving forces for the self-assembly of natural polymers, hydrogen bonding mainly occurs between polar groups such as hydroxyl, carboxyl, and amino groups contained in polymer molecular chains, or between these groups and water molecules. For example, the glucuronic acid units and N-acetylglucosamine units on the hyaluronic acid molecular chain are alternately connected through intramolecular and intermolecular hydrogen bonds to form linear rigid segments, which further crosslink through hydrogen bonds to construct the initial three-dimensional network skeleton [21]. The hydrogen bonds formed between the amino groups in chitosan molecules and water molecules can not only enhance the intermolecular binding force but also improve the hydrophilicity and swelling performance of the gel, enabling the gel network to more easily accommodate drug molecules and bioactive substances [22].

Hydrophobic interaction targets hydrophobic groups contained in polymer molecular chains, such as methyl groups in chitosan molecules and hydrophobic amino acid residues like leucine, isoleucine, and phenylalanine in peptide molecules. In an aqueous environment, these hydrophobic groups spontaneously aggregate to form hydrophobic microdomains to avoid repulsion with polar water molecules, thereby driving the folding, entanglement, and crosslinking of polymer molecular chains and ultimately forming a stable gel network [23]. For instance, aliphatic peptide molecules aggregate through hydrophobic amino acid residues at the N-terminus and C-terminus to form β-sheet or α-helix secondary structures, which further stack and construct a three-dimensional gel with high porosity through hydrophobic interactions [24]. Among synthetic polymers, the polycaprolactone segment of polycaprolactone-polyethylene glycol block copolymers aggregates to form a core region through hydrophobic interactions, while the polyethylene glycol segment extends in the aqueous environment to form “core–shell” structured micelles, which can form self-assembled hydrogels through further aggregation [25]. Electrostatic interaction occurs between polymer molecules with opposite charges or between charged groups on polymer molecular chains, achieving intermolecular crosslinking and aggregation through Coulombic attraction [26]. For example, after mixing cationic chitosan and anionic hyaluronic acid, the two polymer molecules rapidly combine through electrostatic attraction between positive and negative charges to form polyelectrolyte complexes, which then spontaneously assemble into a stable gel network [27]. By adjusting parameters related to polymer charge density such as the degree of deacetylation of chitosan and the degree of carboxyl substitution of hyaluronic acid, the strength of electrostatic interaction can be precisely regulated, enabling flexible adjustment of the gelation time and mechanical strength of the gel.

π-π stacking mainly exists in polymer molecules with aromatic structures, such as tyrosine-modified peptides and benzoboroxole-functionalized synthetic polymers [28]. The π electron clouds in aromatic groups overlap to form weak interactions [29]. Although the strength of this interaction is weak when acting alone, it can significantly enhance the stability of the gel network under the synergistic effect of multiple aromatic groups in the molecular chain [30,31]. For example, in benzoboroxole-functionalized polyethylene glycol polymers, the benzene rings on the molecular chain approach each other through π-π stacking to form locally ordered structures, which can not only promote molecular self-assembly but also improve the response specificity of the gel to reactive oxygen species, providing a structural basis for intelligent drug delivery [32].

Table 1 summarizes the therapeutic effects of hydrogel drug delivery systems driven by different self-assembly mechanisms in animal models of osteoarthritis, offering insights for the rational design of self-assembled hydrogel-based OA therapies.

1. Intermolecular interactions: Under physiological conditions (37 °C, pH 7.2–7.4), the material molecules undergo self-assembly from a dispersed state into linear chains via non-covalent interactions, including hydrogen bonding, hydrophobic interactions, electrostatic forces, and π-π stacking. These chains then further aggregate to form a stable network.
2. Three-dimensional hydrogel formation: The aggregated linear chains construct a transparent, porous three-dimensional hydrogel scaffold, which is equipped with drug encapsulation sites and modification sites for cartilage-targeting ligands (e.g., RGD motifs).
3. Functional output and OA therapeutic relevance: The hydrogel exhibits minimally invasive injectability, controlled degradability, and intelligent responsiveness to pH and reactive oxygen species (ROS). It can be delivered intra-articularly via injection, enabling targeted therapeutic applications for OA.

According to differences in material sources, self-assembled hydrogels can be divided into three categories: natural polymer-based [21], synthetic polymer-based [33], and composite polymer-based [34], each with distinct self-assembly characteristics and functional advantages [35]. Natural polymer self-assembled hydrogels are prepared from natural biological materials such as chitosan [36], hyaluronic acid [37], collagen [38], gelatin, alginate [39], and peptides [40]. Their molecular structures have good similarity to the extracellular matrix of human tissues, thus possessing excellent biocompatibility, biodegradability, and bioactivity [41]. The self-assembly of these materials mostly relies on the synergistic driving force of hydrogen bonding and hydrophobic interaction [42]. For example, collagen molecules spontaneously aggregate through hydrogen bonding and hydrophobic interaction between triple helix structures to form a highly elastic gel network, and the degradation products of the gel such as amino acids and glucosamine can be metabolized and absorbed by the human body without the risk of foreign body residue [43]. Some natural polymers also have inherent pharmacological activities [44]. For instance, hyaluronic acid can supplement synovial fluid components and inhibit the release of inflammatory factors [45], while chitosan has antibacterial properties and promotes tissue repair [46,47], further expanding the therapeutic potential of self-assembled hydrogels.

Synthetic polymer self-assembled hydrogels are made from artificially synthesized polymers such as polyethylene glycol [26,48], polycaprolactone [49], polylactic acid [50], and polyacrylic acid derivatives [51]. Their molecular structures can be precisely designed through chemical synthesis, so their physicochemical properties such as molecular weight, hydrophilic-hydrophobic balance, and degradation rate have high adjustability. The self-assembly of these materials usually relies on hydrophobic interaction and electrostatic interaction. For example, polycaprolactone-polyethylene glycol block copolymers can control the strength of hydrophobic interaction by adjusting the molecular weight of the polycaprolactone segment, thereby achieving precise regulation of the gel degradation cycle ranging from several days to several months [52]. The advantage of synthetic polymers lies in their strong controllability of mechanical strength [53]. The compression and shear resistance of the gel can be improved by introducing crosslinking points or adjusting the block ratio to adapt to dynamic mechanical environments such as the joint cavity [54]. Meanwhile, their high structural stability can effectively protect loaded drugs such as growth factors and nucleic acids from enzymatic hydrolysis or environmental damage [55].

Composite polymer self-assembled hydrogels integrate the advantages of natural polymers and synthetic polymers through physical blending, chemical grafting, or in situ composite methods to achieve synergistic optimization of performance and solve the defects of single-type polymers such as insufficient mechanical strength of natural polymers and lack of bioactivity of synthetic polymers [56,57]. For example, chitosan-polyethylene glycol composite hydrogels not only retain the biocompatibility and antibacterial activity of chitosan [58] but also improve the mechanical strength and degradation resistance of the gel through the introduction of polyethylene glycol [59,60]. At the same time, the hydrophilicity of polyethylene glycol can improve the swelling performance of chitosan, making the gel more suitable for loading water-soluble drugs. Hyaluronic acid-hydroxyapatite composite hydrogels use hyaluronic acid as the gel matrix to provide biocompatibility and hydrophilicity [37,61]. Hydroxyapatite nanoparticles simulating the inorganic components of bone tissue are dispersed in the gel network through hydrogen bonding and electrostatic interaction, which can not only enhance the mechanical strength and cartilage targeting of the gel but also provide calcium and phosphorus ions for cartilage repair through the sustained release of hydroxyapatite, achieving the synergistic effect of “carrier support-nutrient supply-tissue repair”. Such composite hydrogels can flexibly adjust the ratio and composite method of natural and synthetic materials according to specific needs in osteoarthritis treatment, such as enhancing mechanical stability, improving targeting, and extending the degradation cycle, thereby better adapting to the complex dynamic microenvironment and pathological state within the joint.

**Table 1:** Summary of therapeutic effects of hydrogel drug delivery systems with different self-assembly driving mechanisms in animal models of osteoarthritis.

Material Type	Self-Assembly Driving Force	Drug Type	Animal Experiment Effect	Data Source
Chitosan-adipic dihydrazide-hyaluronic acid aldehyde composite hydrogel	Hydrogen bonding, electrostatic interaction	Diclofenac sodium	In rat OA model, the concentrations of TNF-α and IL-1β in synovial fluid decreased by 58.3% and 62.1%, respectively, the histological score of cartilage decreased from 6.8 to 3.1, and the drug retention time was extended to 28 days	[62]
Peptide-hyaluronic acid composite hydrogel	Hydrophobic interaction, hydrogen bonding	Transforming growth factor-β1 (TGF-β1)	In rabbit OA model, the expression of type II collagen in cartilage defect area increased by 73.2%, the content of aggrecan increased by 68.5%, and the cartilage repair rate reached 52.3% at 4 weeks	[63]
Polycaprolactone-polyethylene glycol block copolymer hydrogel	Hydrophobic interaction, temperature-sensitive self-assembly	Dexamethasone	In mouse OA model, the area of synovial inflammatory infiltration decreased by 45.7%, the joint weight-bearing ratio increased from 32% to 65%, and the therapeutic effect lasted for more than 8 weeks	[64]
Alginate-dopamine-regenerated silk fibroin composite hydrogel	Hydrogen bonding, hydrophobic interaction	Mesenchymal stem cell (MSC)-derived exosomes	In rat OA model, the proportion of M2 macrophages increased by 50.2%, the expression of IL-10 increased by 45.3%, and the cartilage defect repair area reached 68.4% at 8 weeks	[65]
Benzoboroxole-functionalized polymer hydrogel	π-π stacking, hydrophobic interaction	Triamcinolone acetonide	In rat OA model, the cumulative drug release rate reached 78.5% under ROS stimulation in the joint within 7 days, the concentration of inflammatory factors decreased by 65.2%, and the degree of cartilage degeneration was significantly reduced	[66]
Carboxymethyl chitosan-polyethylene glycol composite hydrogel	Electrostatic interaction, hydrogen bonding	Diclofenac sodium	In rat OA model (pH 6.8 environment), the drug release rate was 2.5 times higher than that in normal pH environment, the pain score decreased by 60%, and the inhibition rate of cartilage matrix degradation reached 48%	[67]
Collagen-chitosan composite hydrogel	Hydrogen bonding, hydrophobic interaction	Chondroitin sulfate	In rabbit OA model, the viscosity of synovial fluid increased by 2 times, the activity of MMP-13 decreased by 52%, and the degree of cartilage wear was reduced by 50% at 8 weeks	[68]
Thioether-modified polyethylene glycol-polylactic acid block copolymer hydrogel	Hydrophobic interaction, ROS-sensitive self-assembly	Interleukin-1 receptor antagonist (IL-1Ra)	In mouse OA model, the concentrations of inflammatory factors IL-1β and IL-6 in the joint decreased by 63.7% and 58.9%, respectively, and the apoptosis rate of chondrocytes decreased by 42%	[69]
Chitosan-adipic dihydrazide-hyaluronic acid aldehyde composite hydrogel	Hydrogen bonding, electrostatic interaction	Diclofenac sodium	In rat OA model, the concentrations of TNF-α and IL-1β in synovial fluid decreased by 58.3% and 62.1%, respectively, the histological score of cartilage decreased from 6.8 to 3.1, and the drug retention time was extended to 28 days	[70]

### 2.2. Key Properties of Self-Assembled Hydrogels Oriented to OA Treatment

In response to the pathological characteristics of OA and the application scenario within the joint, self-assembled hydrogels need to possess a series of specific key physicochemical and biological properties [71]. In terms of physicochemical properties, their rheological behavior must adapt to the mechanical environment of repeated joint movements [72]. Specifically, the storage modulus should be maintained between ten and one hundred kilopascals, and the ratio of loss modulus to storage modulus should be less than 0.3 to ensure that the gel maintains structural integrity and necessary compliance during joint activities [73]. Meanwhile, the gel should have good injectability, characterized by shear-thinning behavior, with an injection viscosity usually lower than five Pascals-seconds [74], and can quickly recover to a gel state under physiological conditions within one to five minutes to achieve minimally invasive delivery and prevent leakage. In addition, to cope with the continuous shear force caused by joint movement, self-assembled hydrogels usually need to have excellent shear self-healing ability, which can quickly restore their structural integrity after damage [75], for example, achieving a repair efficiency of more than 80% within ten minutes. The precise regulation of the degradation cycle is also crucial, which needs to match the usual treatment cycle of four to twelve weeks to avoid insufficient drug release due to too fast degradation or foreign body reaction due to too slow degradation.

In terms of biological properties, excellent biocompatibility is the primary requirement, which needs to ensure low toxicity to chondrocytes and no significant induction of synovial inflammatory response [76]. To improve therapeutic efficacy and reduce systemic side effects, endowing the gel with cartilage targeting is an important direction, which can be achieved by modifying cartilage-specific ligands such as RGD peptides [77]. In addition, efficient drug-loading capacity is also indispensable, which needs to effectively load and sustainably release different types of therapeutic agents [10], including small-molecule anti-inflammatory drugs, growth factors, and even exosomes, while maintaining their biological activity [78].

### 2.3. Comparative Advantages of Self-Assembled Hydrogels over Traditional Hydrogels

Compared with traditional hydrogels relying on chemical crosslinking or physical crosslinking, self-assembled hydrogels exhibit significant advantages in multiple aspects in OA drug delivery applications. In terms of formation method, the self-assembly process avoids the use of chemical crosslinking agents, thereby reducing potential cytotoxicity by 60% to 80% and eliminating the risk of inflammation caused by residual crosslinking agents [79]. In terms of delivery convenience, it can achieve in situ rapid gelation through minimally invasive injection without complex external triggering conditions, which greatly simplifies the operation and improves patient tolerance [21]. In terms of drug release behavior, self-assembled hydrogels can often achieve longer-acting and intelligent responsive release, for example, extending the release cycle of certain drugs by two to three times [80].

Its excellent biocompatibility is reflected in its good adaptability to the intra-articular environment. Studies have shown that the expression level of inflammatory factors induced by it during in vivo retention is significantly lower than that of traditional hydrogels. More importantly, its inherent dynamically reversible network endows it with shear self-healing ability, enabling it to better maintain structural integrity and the stability of drug delivery function under the continuous shear force of joint movement [81]. Animal model experimental data further support these advantages [82]. For example, in a rat osteoarthritis model, the self-assembled peptide hydrogel loaded with dexamethasone is significantly superior to the traditional chemically crosslinked hyaluronic acid hydrogel in terms of cartilage repair rate and systemic safety. These comparative results indicate that self-assembled hydrogels have more advantages in safety, delivery efficiency, and therapeutic effect, and are more suitable for the needs of local drug delivery for osteoarthritis (Table 2) [83].

## 3. Delivery Applications of Self-Assembled Hydrogels in OA Treatment

With their controllable gelation properties, excellent drug-loading capacity, and good biocompatibility, self-assembled hydrogels have developed various delivery application forms in OA treatment, mainly including single-drug targeted delivery systems, exosome composite synergistic delivery systems, and environment-responsive precise delivery systems. Through different molecular and cellular mechanisms, these systems aim to effectively inhibit joint inflammation, protect and repair cartilage, thereby providing diverse potential solutions for OA treatment (Table 3).

### 3.1. Intra-Articular Injectable Self-Assembled Hydrogels (Single-Drug Delivery)

Intra-articular injectable self-assembled hydrogels are currently the most direct and well-studied application form [108]. By loading a single therapeutic drug, this system aims to achieve long-acting targeted treatment of the joint, effectively avoiding the side effects caused by systemic administration and the rapid clearance of drugs in the joint cavity [109,110]. Commonly loaded drugs are mainly classified into three categories: anti-inflammatory drugs such as diclofenac sodium and dexamethasone [111], which are mainly used to inhibit inflammatory response and relieve pain; cartilage protectants such as hyaluronic acid and chondroitin sulfate, which aim to supplement synovial fluid components and delay matrix degradation [112]; and growth factors such as transforming growth factor β1 and bone morphogenetic protein 2, which are expected to promote the synthesis and repair of cartilage matrix [76].

The core mechanism of this delivery system lies in “physical barrier combined with degradation-controlled release”. After minimally invasive injection into the joint cavity, self-assembled hydrogels can rapidly form a three-dimensional network structure under physiological conditions, acting as a physical barrier to limit drug diffusion and significantly extend drug retention time [113]. As the gel network slowly degrades through enzymatic hydrolysis or hydrolysis, the encapsulated drugs are continuously and stably released to the local joint, maintaining effective therapeutic concentrations. A large number of animal experimental data have confirmed the efficacy of this system [114]. For example, in a rat OA model, the temperature-sensitive self-assembled hydrogel loaded with diclofenac sodium can significantly reduce the concentrations of TNF-α and IL-1β in synovial fluid and reduce synovial inflammatory infiltration; while the peptide hydrogel loaded with TGF-β1 can effectively increase the expression of type II collagen and aggrecan in the cartilage defect area [115], improve the histological score of cartilage, and alleviate pain-related behaviors [104].

### 3.2. Self-Assembled Hydrogel-Exosome Composite Synergistic Delivery Systems

As key nanocarriers for intercellular communication, exosomes are rich in various biologically active miRNAs and cytokines, and have the potential to regulate immune responses and promote tissue repair. However, they have a short in vivo half-life and are easily rapidly cleared [116]. The construction of a delivery system by combining exosomes with self-assembled hydrogels can achieve the enhanced effect of “carrier protection and biological activity synergy” [117].

The key to constructing this composite system is to achieve stable loading and controlled release of exosomes [118]. Usually, physical encapsulation is used to wrap exosomes in the three-dimensional network of hydrogels, or specific ligands are modified on the surface to enhance binding stability [119]. Natural polymer-based hydrogels with excellent biocompatibility, such as alginate-dopamine or chitosan-hyaluronic acid composites, are preferred to provide a mild microenvironment for exosomes to maintain their activity [120]. The synergistic effect of this system is reflected in two aspects: on the one hand, the hydrogel, as a protective carrier, can significantly extend the retention time of exosomes in the joint cavity [121]; on the other hand, the continuous release of exosomes combined with the physical support effect of the hydrogel itself can synergistically regulate the polarization of macrophages to the anti-inflammatory phenotype, inhibit chondrocyte apoptosis, and promote cartilage matrix synthesis [122]. Animal studies have shown that such composite delivery systems are significantly superior to single-component treatments in inhibiting inflammation and promoting cartilage repair.

### 3.3. Environment-Responsive Precise Delivery Systems of Self-Assembled Hydrogels

In response to the characteristics of increased reactive oxygen species (ROS) levels and decreased pH value in the OA joint microenvironment [107], environment-responsive self-assembled hydrogel delivery systems have emerged, aiming to achieve on-demand drug release [40], thereby improving the precision and efficiency of treatment. ROS-responsive hydrogels introduce ROS-sensitive groups such as thioether bonds or boronate ester bonds into their molecular chains, which remain stable in the normal environment but undergo chemical bond cleavage under the high ROS environment at the inflammatory site, leading to gel network dissociation and rapid drug release [123]. For example, hydrogels based on benzoboroxole-functionalized polymers can achieve targeted triggered drug release under ROS stimulation.

PH-responsive hydrogels adopt pH-sensitive polymers such as polyhistidine or carboxymethyl chitosan, whose self-assembly behavior changes with the environmental pH, and depolymerizes to release drugs in the acidic inflammatory microenvironment [124]. Research cases have shown that the ROS-responsive hydrogel loaded with triamcinolone acetonide can intelligently release drugs according to the degree of inflammation in a rat model of OA [66], significantly reducing the level of inflammatory factors and alleviating cartilage degeneration, and its efficacy is superior to that of the non-responsive control group. Similarly, the pH-responsive carboxymethyl chitosan-polyethylene glycol hydrogel loaded with diclofenac sodium exhibits accelerated drug release behavior in a simulated acidic OA environment, achieving dynamic regulation of drug administration according to the inflammatory state, thereby optimizing pain control and cartilage repair effects.

## 4. Clinical Translation Challenges and Optimization Directions of Self-Assembled Hydrogels

Self-assembled hydrogels have shown significant application potential in the treatment of knee osteoarthritis. However, their translation from basic research to clinical practice still faces severe challenges in terms of technical feasibility, long-term safety, and regulatory systems.

Currently, most relevant research on self-assembled hydrogels is at the preclinical stage, and their effectiveness has primarily been validated in animal models. Globally, no hydrogel product based on a strict self-assembly mechanism has been officially approved for the treatment of OA [125]. It is worth noting that some injectable hydrogels employing temperature-sensitive or ionic crosslinking mechanisms have entered Phase I or Phase II clinical trials. For example, a temperature-sensitive self-assembled hydrogel based on a polycaprolactone-polyethylene glycol block copolymer developed by a biotechnology company has completed Phase I clinical trials. This study enrolled 20 patients with mild to moderate knee OA, and the results showed that intra-articular injection was safe with no serious adverse reactions, intra-articular retention time reached 4 weeks, and patients’ pain scores decreased by 35% compared to baseline. Another chitosan-hyaluronic acid composite self-assembled hydrogel is in Phase II clinical trials, aiming to evaluate its therapeutic effect when loaded with mesenchymal stem cell-derived exosomes [126]. Preliminary data indicate it can significantly reduce levels of joint inflammatory factors. Although these products differ from the systems focused on in this review in terms of self-assembly driving mechanisms, their technical approaches for intra-articular delivery and sustained release remain valuable references.

Technical challenges are first reflected in the consistency of large-scale production processes. The self-assembly process is highly sensitive to environmental parameters such as temperature, pH, and ionic strength [18]. During process scale-up, key quality attributes like gelation time, mechanical properties [127], and drug-loading efficiency are prone to batch-to-batch variations, with fluctuations ranging from 15% to 20% [128], directly affecting product quality controllability and therapeutic reliability. Secondly, the complex biomechanical environment of the knee joint poses a severe test to the long-term stability of the material. Natural polymer-based hydrogels are susceptible to structural fatigue or even damage under continuous shear forces generated by repeated joint movements, which may lead to premature drug release [129]. Simultaneously, the dilution effect of synovial fluid and the various enzymes it contains may accelerate the degradation of the gel network [130]. Additionally, the encapsulation efficiency of existing drug-loading systems for hydrophobic small-molecule drugs is generally low, with some systems even below 70%, and the compatibility between drug molecules and carrier materials may interfere with the self-assembly process, resulting in an unstable gel structure [131].

Regarding safety, the current evaluation system remains incomplete. Most studies are based only on short-term animal experiments lasting four to twelve weeks, lacking systematic assessment of chronic biological reactions caused by long-term material retention (more than six months). This makes it difficult to fully evaluate potential risks such as chronic inflammation, immunogenicity, and tissue adhesion [132]. Precise control of material degradation behavior remains a major challenge: too rapid degradation may lead to insufficient drug release and failure to achieve the intended therapeutic effect; too slow degradation may trigger foreign body reactions and even exacerbate local inflammation [80]. Particularly for synthetic polymer hydrogels, the in vivo metabolic pathways, tissue accumulation tendencies, and potential long-term toxicity of their degradation products still require clarification through more in-depth toxicological studies [133].

There is also a noticeable gap in regulatory science. Currently, commonly used OA animal models, such as collagenase-induced models or surgical instability models [134,135], have inherent differences in pathological characteristics from the natural progression of human disease, making it difficult to directly extrapolate preclinical efficacy data to humans. More importantly, for such novel delivery systems, specialized review guidelines and technical standards are lacking. Evaluation methods and qualification criteria for key attributes such as gelation characteristics, drug release kinetics [136], and long-term biosafety have not been unified, significantly increasing the complexity of product registration applications and technical reviews.

To promote clinical translation, collaborative optimization in technology, safety, and regulation is necessary. Technical optimization should focus on establishing standardized production processes. For example, combining microfluidic technology with automated control can achieve precise regulation of key self-assembly parameters and control the batch-to-batch coefficient of variation within 5% [137]. Material modification, such as introducing reinforcing phases like hydroxyapatite nanoparticles to improve mechanical properties [138,139], or incorporating amphiphilic structures through molecular design to enhance the loading capacity for hydrophobic drugs, is an important approach to improving performance [140]. Intelligent design has become a significant trend. By integrating ROS-responsive, pH-responsive, or enzyme-responsive elements, on-demand drug release at the lesion site can be achieved [141].

The construction of a safety evaluation system needs to be systematic and long-term. It is essential to conduct long-term animal retention experiments lasting six months to one year to comprehensively investigate the in vivo degradation behavior of materials, the metabolic fate of degradation products, and potential organ toxicity. At the same time, a standardized safety evaluation paradigm covering multiple dimensions such as cytotoxicity, genotoxicity, local tissue reactions, and systemic toxicity should be established [142].

The clinical translation pathway should adopt a gradual strategy. First, small-sample, first-in-human trials should be conducted, focusing on investigating injection safety, local tolerance, and basic pharmacokinetic characteristics. Based on this, proof-of-concept Phase II clinical trials should be promoted to obtain preliminary efficacy evidence. Early communication and cooperation between industry and drug regulatory authorities are crucial, and joint efforts should be made to formulate quality analysis methods and evaluation standards suitable for such innovative products (Table 4) [143].

With the deep integration of materials science, pharmaceutics, and clinical medicine, the next generation of self-assembled hydrogel systems will develop towards functional integration and intelligence. By temporally controlling the release kinetics of different therapeutic drugs such as anti-inflammatory agents and growth factors, synergistic treatment combining anti-inflammation and repair is expected to be achieved. Integrated diagnostic and therapeutic systems with imaging and tracking functions provide the possibility for real-time monitoring and efficacy evaluation during treatment. Although no mature products are on the market yet, the profound technical accumulation in this field has laid a solid foundation for clinical translation. It is expected that within the next three to five years, multiple OA treatment products based on self-assembled hydrogel technology will enter the clinical development stage, promising to provide patients with more precise, long-acting, and minimally invasive new treatment options.

## 5. Conclusions

Self-assembled hydrogels have formed significant technical advantages in the field of OA drug delivery due to their unique molecular self-assembly mechanisms and tunable functional properties. Such materials can achieve in situ gelation without the involvement of chemical crosslinking agents or external physical induction, which is highly consistent with the technical requirements of minimally invasive treatment for delivery systems; their precisely controllable degradation kinetic characteristics provide an ideal carrier platform for sustained and controlled drug release; their excellent biocompatibility effectively avoids potential in vivo safety risks of traditional treatment methods. Through diversified advanced modes such as single-drug targeted delivery, exosome composite synergistic delivery, and environment-responsive precise delivery, self-assembled hydrogels can precisely intervene in key pathological links such as inflammatory cascade reaction, cartilage matrix degradation, and tissue repair disorders targeting the complex pathological process of OA, achieving synergistic therapeutic effects of anti-inflammatory effect, cartilage protection, and tissue repair, opening up a new technical path to break through the inherent limitations of existing treatment methods, and demonstrating broad clinical application prospects.

However, the translation of self-assembled hydrogels from the concept verification stage to clinical practical application still faces multiple key bottlenecks. At the technical level, the control of batch-to-batch consistency during large-scale production, the maintenance of long-term structural stability of materials under the dynamic biomechanical environment of the joint, and the improvement of efficient loading capacity of hydrophobic therapeutic drugs are all core technical problems that need to be solved urgently. At the safety level, systematic long-term biocompatibility evaluation, personalized matching of degradation behavior with the pathological progress of individual patients, and evaluation of the metabolic pathways and potential toxicity of degradation products in the body still need to be fully clarified through rigorous scientific research. At the regulatory and translation level, the translation gap between preclinical animal model data and human efficacy, as well as the lack of special evaluation standards and review paths for such novel delivery systems, together constitute the main restrictive factors for the translation of achievements into clinical products. The effective solution to the above challenges relies on the in-depth collaborative research of multiple disciplines such as materials science, biomedical engineering, pharmacology, and clinical medicine.

Looking forward to the future, the research and development of the next generation of self-assembled hydrogels will focus on two core directions: precise regulation of performance and integration of multi-functional systems. Through in-depth molecular design and optimization of synthetic processes, precise customization of key material parameters such as mechanical strength and degradation rate can be achieved to meet the personalized treatment needs of OA patients at different disease stages. With the construction of advanced targeted ligand modification technology and intelligent response mechanisms, the targeting specificity of the system to pathological cartilage tissue and the release precision of drugs in the spatiotemporal dimension can be further improved. Further integrating multi-drug sequential synergistic delivery strategies, and even integrating diagnostic and therapeutic functions to construct an intelligent integrated diagnosis and treatment system, represents the cutting-edge development trend of this field. With the continuous deepening of relevant basic research, the continuous innovation of preparation processes, and the gradual accumulation of clinical verification data, self-assembled hydrogels are expected to develop into a core technical platform for precise, long-acting, and minimally invasive treatment of OA, significantly improving patients’ quality of life and bringing revolutionary breakthroughs to the field of OA treatment.

## Figures and Tables

**Table 2:** Comparison of Key Properties between Self-Assembled Hydrogels and Traditional Chemically Crosslinked Hydrogels.

Comparison Dimension	Self-Assembled Hydrogels	Traditional Chemically Crosslinked Hydrogels	Advantage Differences
Formation Mechanism	Self-assembly mediated by non-covalent interactions, no chemical crosslinkers required [84]	Covalent bond linkage mediated by chemical crosslinkers [85]	No residual crosslinkers, reducing cytotoxicity by 60–80%
Injectability	Shear-thinning property, in situ gelation after minimally invasive injection (1–5 min) [86,87]	Mostly preformed hydrogels or requiring ex vivo crosslinking, poor injectability [88]	Simplified operation process, significantly improved patient tolerance
Drug Release Duration	Sustained drug release for 2–8 weeks (up to 28 days in some systems) [89]	Rapid drug release (usually completed within 1–2 weeks) [90]	Prolonged drug release cycle by 2–3 folds, reducing administration frequency
Mechanical Self-Healing Ability	Shear self-healing efficiency exceeding 80% (within 10 min) [91]	No mechanical self-healing ability, prone to structural damage under stress [92]	Better adaptation to the dynamic mechanical environment of joints, superior structural stability
Biocompatibility	Significantly reduced expression levels of inflammatory factors [93]	Prone to inducing local chronic inflammatory responses [94]	Reduced joint tissue irritation, lowering the risk of foreign body reactions

**Table 3:** Summary of Applications and Effects of Different Types of Drug Delivery Systems in the Treatment of Osteoarthritis.

Serial Number	Delivery System Type	Representative Material/Combination	Loaded Drug/Active Ingredient	Result	Reference
1	Single-drug Targeted Delivery System	Mesoporous Silica Nanoparticles (MSNs)/Self-healing Hydrogel Composite Cotton Patches	Colchicine	Achieved efficient local anti-inflammatory and cartilage protective effects in an osteoarthritis model, with good skin biosafety.	[95]
2	Single-drug Targeted Delivery System	Adenosine Triphosphate (ATP)/α-Cyclodextrin Host-Guest Inclusion Complexes (PPRs)	Diclofenac Sodium (DS)	Preliminary in vivo treatment in a rat model of acute inflammation showed that ATP hybrid hydrogel has a sustained anti-inflammatory effect.	[96]
3	Single-drug Targeted Delivery System	Oxidized Dextran (Dex-ox), Gelatin, Hyaluronic Acid (HA) Composite System	Naproxen, Dexamethasone	The macroscopic severity of knee osteoarthritis treated with IHDDS was lower, and cartilage preservation was better.	[97]
4	Microenvironment-responsive Precision Delivery System	Sodium Alginate-3-Aminophenylboronic Acid (SA-PBA) Precursor (Sodium Alginate (SA), 3-Aminophenylboronic Acid (PBA)), Tea Polyphenols, Inorganic Composite Nanoparticles, Gluconolactone (GDL)	Triptolide (TPL)	Showed significant inflammation inhibition and optimal regeneration of articular cartilage in a rat model of rheumatoid arthritis (RA).	[98]
5	Exosome Composite Synergistic Delivery System	Poloxamer 407/188 Composite System	Platelet-Rich Plasma-Derived Exosomes (PRP-Exo)	Exo-Gel increased the retention of exosomes in local joints, inhibited chondrocyte apoptosis and hypertrophy, and delayed the development of subtalar osteoarthritis (STOA).	[99]
6	Exosome Composite Synergistic Delivery System, Microenvironment-responsive Precision Delivery System	Poloxamer F-127/Hyaluronic Acid (HA) Composite System	Primary Chondrocyte-Derived Exosomes	It can enhance chondrocyte functions related to cartilage repair. The sustained release of exosomes from the hydrogel can achieve long-term cartilage protective effects.	[77]
7	Microenvironment-responsive Precision Delivery System	Formaldehyde-Glycerol-Borax Composite System	Dexamethasone	In a mouse model of osteoarthritis induced by destabilization of the medial meniscus (DMM), the DLTH system effectively reduced OA-related bone destruction, improved synovitis symptoms, and delayed disease progression.	[100]
8	Exosome Composite Synergistic Delivery System	Mannose Oligosaccharide (MOS)-Modified Chondroitin Sulfate/Hyaluronic Acid Composite System	Macrophage Extracellular Vesicles (EVs)	The released extracellular vesicles (EVs) showed significant support for the formation and preservation of new cartilage.	[101]
9	Single-drug Targeted Delivery System	Thiol-Modified Hyaluronic Acid (HA-SH), NO Scavenging/H_2_S Releasing Copolymer (DNRS Copolymer)	Methotrexate (MTX)	The MTX-loaded hydrogel (MTX/DNRS gel) showed significant inflammation inhibition effect in a rat model of collagen-induced arthritis after intra-articular injection, effectively improving clinical symptoms; at the same time, it promoted bone erosion repair, restored the microenvironmental stability of the lesion site, and significantly enhanced MTX therapeutic efficacy.	[102]
10	Exosome Composite Synergistic Delivery System	Methacrylated Hyaluronic Acid (HAMA), Methacrylated Gelatin (GelMA), Photoinitiator (LAP)	Stem Cells and Their Derived Exosomes (Exo)	The exosomes released from the carrier particles further enhanced cartilage repair efficacy through synergistic effects.	[103]
11	Microenvironment-responsive Precision Delivery System	Methacrylated Hyaluronic Acid (HAMA), MMP13 Substrate Peptide (MMP13sp), Cationic Liposomes	Celecoxib	In a rat model of osteoarthritis induced by anterior cruciate ligament transection (ACL) combined with partial medial meniscectomy, the articular cartilage degradation process in the HAMA/MMP13sp/Lipo@Celecoxib treatment group was significantly improved.	[104]
12	Exosome Composite Synergistic Delivery System	Decellularized Cartilage Matrix (HECM) Microgels	Self-assembled Bifunctional Exosomes	The system prolonged the retention time of exosomes in vivo. It helps promote the repair of damaged cartilage. By regulating the PI3K/AKT signaling pathway, it slowed down the progression of osteoarthritis.	[105]
13	Microenvironment-responsive Precision Delivery System	Methacrylated Gelatin (GM) Hydrogel Microspheres, Novel Liposome WYRGRL-DOTAP-Lipo	Kartogenin (KGN)	In a mouse OA model, intra-articular injection of WDLKG significantly inhibited cartilage degradation and confirmed the sustained release of WDLKG.	[106]
14	Microenvironment-responsive Precision Delivery System	Short Peptide Hydrogel	Insulin-like Growth Factor 1 (IGF-1)	The constructed short peptide hydrogel has excellent physicochemical properties and biological functions, and can achieve dual functions of reactive oxygen species (ROS)-responsive drug delivery and ROS scavenging.	[107]

**Table 4:** Clinical Translation Challenges and Optimization Strategies of Self-Assembled Hydrogels for OA Treatment.

Clinical Translation Challenges	Specific Manifestations	Core Optimization Strategies	Expected Outcomes
Consistency in Large-Scale Production	Batch-to-batch fluctuations of key indicators (gelation time, mechanical strength, drug-loading efficiency) reaching 15–20%	Integrating microfluidic technology with automated control systems to precisely regulate key self-assembly parameters [144]	Batch coefficient of variation controlled within 5% [145]
Long-Term Stability in Joints	Dynamic mechanical shear-induced material structural fatigue, and synovial fluid dilution accelerating gel degradation	Incorporating hydroxyapatite nanoparticles to enhance mechanical strength; optimizing polymer block ratios [146]	Prolonged in vivo retention time of hydrogels to 4–8 weeks, matching OA treatment cycles [147]
Hydrophobic Drug-Loading Efficiency	Low loading efficiency (<70%) for hydrophobic drugs in some systems; drug-carrier compatibility interfering with self-assembly	Introducing amphiphilic structures via molecular design; adopting composite polymer carrier systems [148]	Hydrophobic drug-loading efficiency increased to over 85%, with significantly improved gel structural stability [149]
Insufficient Long-Term Safety Evaluation	Most existing studies are short-term animal experiments (4–12 weeks), lacking data on chronic biological reactions induced by long-term material retention	Conducting long-term animal retention experiments (6–12 months) to systematically evaluate material degradation behavior, metabolic pathways of degradation products, and potential toxicity [150]	Clarified biosafety of degradation products, reducing risks of chronic inflammation and immunogenicity [151]
Lack of Regulatory Standards	Absence of exclusive review guidelines for such novel delivery systems; ununified evaluation methods for key attributes (e.g., drug release kinetics, biosafety) [152]	Promoting industry-regulatory authority collaboration to develop dedicated quality analysis methods and evaluation standards; adopting a phased strategy for Phase I/II clinical trials [153]	Simplified product registration process, accelerating technical clinical translation

## Data Availability

No new data were created or analyzed in this study. Data sharing is not applicable to this article.
